# Using PAR4 Inhibition as an Anti-Thrombotic Approach: Why, How, and When?

**DOI:** 10.3390/ijms20225629

**Published:** 2019-11-11

**Authors:** Simeng Li, Volga Tarlac, Justin R. Hamilton

**Affiliations:** Australian Centre for Blood Diseases, Monash University, Melbourne VIC 3800, Australia; sli380@student.monash.edu (S.L.); Volga.Tarlac@monash.edu (V.T.)

**Keywords:** protease-activated receptors, thrombin, platelet, thrombosis, antagonists, anti-thrombotics, anti-platelets

## Abstract

Protease-activated receptors (PARs) are a family of four GPCRs with a variety of cellular functions, yet the only advanced clinical endeavours to target these receptors for therapeutic gain to date relates to the impairment of platelet function for anti-thrombotic therapy. The only approved PAR antagonist is the PAR1 inhibitor, vorapaxar—the sole anti-platelet drug against a new target approved in the past 20 years. However, there are two PARs on human platelets, PAR1 and PAR4, and more recent efforts have focused on the development of the first PAR4 antagonists, with first-in-class agents recently beginning clinical trial. Here, we review the rationale for this approach, outline the various modes of PAR4 inhibition, and speculate on the specific therapeutic potential of targeting PAR4 for the prevention of thrombotic conditions.

## 1. Introduction

**Figure 1:**
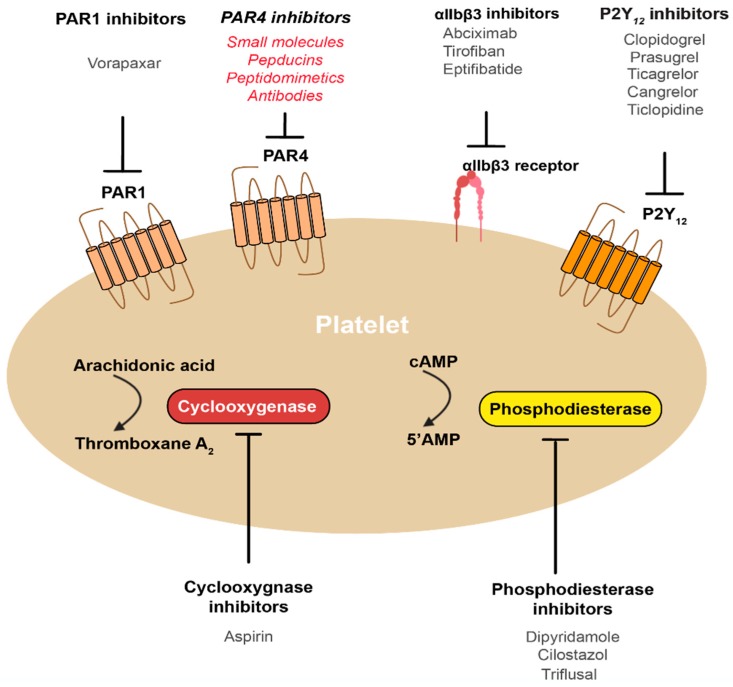
Currently available anti-platelet agents (black text) target five distinct platelet proteins: Cyclooxygenase (aspirin), P2Y_12_ (clopidogrel, prasugrel, ticlopidine, ticagrelor, cangrelor), α_II_bβ_3_ (abciximab, tirofiban, eptifibitide), phosphodiesterase (dipyridamole, cilostazol, triflusal) or PAR1 (vorapaxar). PAR4 is an emerging target for anti-platelet drugs, with a number of different strategies to inhibit the receptor currently being pursued, as indicated (red italicised text).

Cardiovascular disease is the leading cause of death in the world, predominantly because of the prevalence of heart attacks and occlusive strokes. The rupture of atherosclerotic plaques is the major trigger of these two conditions, but it is the thrombotic response to this plaque rupture that ultimately causes the end-stage disease. Platelets are the essential component of this thrombotic response, and so drugs that inhibit platelet function in this setting are the primary pharmacotherapy for the prevention of such cardiovascular diseases. All currently available anti-platelet agents block one of five target proteins in the platelet: The intracellular signalling enzymes cyclooxygenase (aspirin) or phosphodiesterase (e.g., dipyridamole), the cell surface G protein-coupled receptors P2Y_12_ (e.g., clopidogrel) or PAR1 (vorapaxar), or the integrin α_IIb_β_3_ (e.g., abciximab) (Figure 1). Despite five distinct drug targets, all of the currently available anti-platelet agents have shortcomings that limit their clinical efficacy and/or utility. For example, aspirin and P2Y_12_ receptor antagonists are the leading agents for long-term preventative therapy yet prevent fewer than 20% of recurrent thrombotic events even when used in combination [1], phosphodiesterase inhibitors have a number of problematic side effects, such as arrhythmia [2], the αIIbβ3 antagonists all require intravenous administration and cause substantial bleeding, which precludes their use as long term preventatives [3,4,5], and vorapaxar causes an unacceptably high bleeding risk in several patient groups when administered in combination with aspirin and/or clopidogrel [6,7,8]. These limitations have driven ongoing efforts to identify new targets for anti-platelet drugs that have the potential to improve on efficacy and/or provide fewer side effects, particularly on bleeding. PAR4 is one such target that has received substantial recent attention and has advanced to a clinical trial. Here, we review the rationale for this approach (why?), outline the various modes of PAR4 inhibition (how?), and speculate on the specific therapeutic potential of targeting PAR4 for the prevention of thrombotic conditions (when?).

## 2. What is the Function of PAR4 on Platelets?

The PARs belong to the superfamily of seven transmembrane spanning G protein-coupled receptors [9]. There are four PARs: PAR1, PAR2, PAR3 and PAR4. These receptors are distinguished by their unique mechanism of activation that involves proteolytic cleavage of the receptor’s amino terminus and de-encrypting of a “tethered ligand” which self-activates the receptor via intramolecular binding [10]. These receptors are widely expressed in cells and tissues, and respond to a number of proteases. However, in the context of platelet biology, PARs are largely considered as receptors for coagulation proteases, with thrombin being the most potent and, arguably, relevant activator of platelet PARs. Human platelets express PAR1 and PAR4, and both receptors respond to thrombin. Yet different species express different sets of PARs on their platelet surface. For example, guinea pigs express PAR1, PAR3 and PAR4 [11], while mice and rats express PAR3 and PAR4 [12,13]. Indeed, it appears that only primates express the repertoire of PAR1 and PAR4 on platelets—a fact that has significantly hampered investigations into platelet PAR function and the impact of their inhibition.

**Figure 2:**
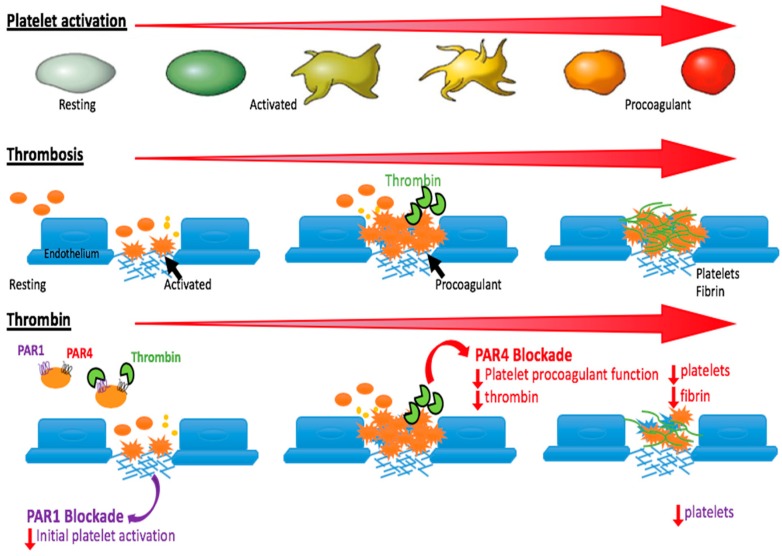
Top: Increasing platelet activation, moving from resting platelets (unactivated; gray) through to initial shape change (yellow) and ultimately PS-exposing procoagulant platelets (red). Middle: Increasing extent of thrombosis correlates with increasing platelet activation. When a blood vessel gets injured, platelets adhere to the site of injury through the binding of exposed subendothelial proteins in the vasculature and glycoprotein receptors on the platelet. Adhesion triggers initial platelet activation, shape change and release of molecular contents. Following activation, phosphatidylserine is exposed on the platelet surface, and these platelets become procoagulant, resulting in further thrombin generation and platelet activation. Bottom: Effects of blocking PAR1 (early stage platelet activation; purple) versus PAR4 (late stage platelet activation; red). Initial platelet function is driven by low thrombin concentrations and PAR1 activation. Later platelet responses, including procoagulant platelet function, is driven by PAR4. Reproduced with permission from Reference [31].

For the first decade or so of platelet PAR research, the focus was squarely on PAR1. For much of this time, PAR4 was considered to be a “backup” receptor—in part because PAR4 requires higher thrombin concentrations for activation and induces a slower signalling response [14]. This decreased sensitivity of PAR4 to thrombin is likely due to structural differences between the two receptors. Specifically, PAR1 contains a hirudin-like thrombin-binding site that is absent in PAR4 [15]. Instead, PAR4 contains an anionic sequence downstream of the thrombin cleavage site that appears to be important for allowing a sustained thrombin signal by PAR4 [16]. This results in distinct signalling kinetics of the two receptors: PAR1 activation drives a rapid initial signal, whereas PAR4 activation induces a slower, but more prolonged response. In platelets, such distinct signalling appears to underlie distinct functions. In particular, PAR1 activation appears to drive early platelet responses, such as initial α_IIb_β_3_ activation and secretion events, while PAR4 is predominantly responsible for the sustained platelet-secretion kinetics [17] and platelet procoagulant function [18]. This is not surprising given that these later responses are known to be heavily reliant on sustained, elevated, intracellular calcium levels [18,19]. Indeed, selective activation of PAR4 releases coagulation factor V from α-granules [20], microparticle shedding [18], sustained Akt phosphorylation [21], and phosphotidylserine (PS) exposure on the platelet surface [18,22]. Although selective activation of PAR1 is also capable of inducing several of these responses, suggesting a level of redundancy in PAR-mediated platelet signalling [23,24], inhibition of PAR4 appears a more effective strategy to impair these later-stage platelet events than inhibition of PAR1 [18,22]. This increased efficacy of PAR4 antagonism may provide a distinction over targeting PAR1 by acting at a later time and place and by interfering with unique platelet functions (Figure 2).

## 3. Why Target PAR4 for Anti-Thrombotic Therapy?

What is the rationale for targeting PAR4 over PAR1? Firstly, PAR1 inhibition has some major clinical limitations as an anti-thrombotic approach. Vorapaxar is a competitive PAR1 antagonist that was recently approved for the prevention of myocardial infarction and peripheral arterial disease [7,25]. Vorapaxar is a reversible inhibitor that binds at or near the tethered ligand binding site within the second extracellular loop of the receptor [26]. It is highly selective and displayed no effect on other agonist-induced platelet aggregation responses in pre-clinical testing [6,27,28]. Vorapaxar was studied in two phase III trials: The Thrombin Receptor Antagonists for Clinical Event Reduction in acute coronary syndrome (TRACER) trial and the TRA 2P-TIMI trial [8,29]. Vorapaxar was approved by the FDA in 2014 for the prevention of myocardial infarction and for the treatment of peripheral arterial disease. However, results from these trials revealed increased severe bleeding rates in patients receiving vorapaxar in addition to standard-of-care, most notably, including intracranial haemorrhage in patients with a prior stroke [30]. This bleeding risk has substantially limited the clinical use of vorapaxar. As a result, much focus has shifted to PAR4. One major strength in the rationale for targeting PAR4 over PAR1 is the distinct functions of the two platelet thrombin receptors, as outlined above. This provides an important point of difference between drugs that block PAR4 function and all other anti-platelet agents, including PAR1 antagonists, such as vorapaxar. Specifically, all other agents broadly act at the same time and place by predominantly blocking early events of platelet activation and deposition in the setting of thrombosis. In contrast, targeting PAR4 is more effective at blocking later-stage platelet events, such as slower platelet secretion events and PS exposure and its subsequent effect on platelet procoagulant activity (Figure 2) [18,22]. This discrepancy is borne out in experimental systems, as well as recent clinical data. For example, selective PAR4 inhibition in human isolated platelets prevents the induction of platelet procoagulant activity, whereas selective inhibition of PAR1 does not [18]. Along similar lines, patients treated with the PAR1 inhibitor vorapaxar exhibit decreased markers of early platelet activation, but markers of platelet procoagulant activity remain unaffected [27]. Whether or not the predicted effects on these markers of coagulation will be observed following treatment of patients with a PAR4 inhibitor remains to be seen. Regardless, all of the initial pre-clinical and clinical data utilising PAR4 antagonists indicate a broader therapeutic window than standard-of-care, due largely to decreased bleeding. This is discussed in more detail in Section 4, below.

## 4. How Can We Best Target PAR4?

There are a number of different approaches to inhibit PAR4. Current inhibitors can be divided into four classes: Peptidomimetics, pepducins, small molecules and antibodies (see Table 1 for summary).

Peptidomimetics were the first class of PAR4 inhibitors. This approach utilises an inactive analogue of the receptor’s tethered ligand to bind to and block the native ligand and activation method [32,39]. These synthetic peptide blockers were important first steps toward inhibitor development, but have not been pursued clinically, due to the limiting pharmacodynamics of such agents. Pepducins are also peptide-based inhibitors that function by blocking the PAR4:G-protein interaction, thus, blocking subsequent signalling pathways [34,40], although how such an approach will ensure specificity remains unknown [41,42,43] and has also limited clinical development.

The leading small molecule PAR4 inhibitors have come from the program at Bristol-Myers Squibb, with BMS-986120 and BMS-986141 reaching clinical trial [36]. BMS-986120 is an orally active, reversible, small molecule PAR4 inhibitor [36]. It exhibits high binding affinity to PAR4 (K_d_ = 0.098 nM) and appears highly selective [14,44]. BMS-986120 inhibits thrombin-induced aggregation of human platelets [36,45] and in vivo studies in non-human primates revealed impressive efficacy and an improved level of safety (assessed by bleeding) of BMS-986120 versus clopidogrel [14]. BMS-986120 underwent the phase I parallel-group PROBE trial, involving forty participants given BMS-986120 (60 mg) or aspirin (600 mg) followed by aspirin (600 mg) and clopidogrel (600 mg) 18 h after the initial dose. BMS-986120 was well tolerated in all participants, and no significant bleeding or other serious adverse events were reported. Pharmacokinetic studies revealed a half-life of approximately 4 h, while pharmacodynamics were assessed using ex vivo platelet activation, aggregation and thrombus formation assays at 2 and 24 h after drug administration and showed that BMS-986120 induced strong and persistent inhibition of PAR4-mediated platelet activation [36]. An analogue of BMS-986120, BMS-986141, has also been pursued clinically and appears favoured, due to its pharmacokinetic profile. A phase I clinical trial of 148 healthy participants assessed pharmacokinetics, pharmacodynamics, and safety. Participants were given either oral BMS-986141 or aspirin (single and ascending dose) to assess the tolerability and safety of BMS-986141 in the absence and presence of co-administered aspirin (NCT02341638). One very small phase II trial was subsequently conducted. Here, 16 participants who had recently had a mini-stroke and were to receive aspirin as a standard-of-care were given aspirin (75 to 162 mg) plus one of a placebo, BMS-986141 at 0.8 mg, or BMS-986141 at 4.8 mg, although this study was not completed (NCT02671461). Clearly, further clinical study of these candidates is required.

More recently, antibody-based PAR4 blockade has been developed. Early experimental agents revealed that antibodies that bind to defined regions of PAR4 could limit or prevent thrombin-induced PAR4 activation [22,38]. Antibodies targeting the anionic cluster near the thrombin cleavage site on the receptor have been shown to delay thrombin-induced platelet aggregation and in vivo thrombosis [38]. However, antibodies directed against the thrombin cleavage site of PAR4 appear the most effective [18,22]. In 2018, the first monoclonal functional blocking antibody against human PAR4 was reported [22]. This antibody inhibits thrombin-induced aggregation in a highly specific manner and is an effective anti-thrombotic in human blood, suggesting that this approach has potential as an anti-platelet therapy, although these agents have not yet been tested in vivo.

## 5. When Can We Best Target PAR4?

Having defined the mechanism by which PAR4 acts on platelets in the setting of thrombosis and then developed effective and specific antagonists of the receptor suitable for anti-thrombotic therapy, the next question to address is: When would this approach work best? Several options appear likely.

(1) Acute stent thrombosis following percutaneous coronary intervention (PCI) is a rare, but devastating event. Anticoagulation (e.g., with unfractionated heparin or enoxaparin) is used in patients undergoing PCI, and P2Y_12_ inhibitors, such as prasugrel or clopidogrel are generally given in the aftermath of PCI for thrombosis prevention. Given the well-defined role of thrombin and platelets in stent thrombosis [26], PAR4 inhibition may be useful in this setting. However, since patients with stent implantation are generally well managed with existing approaches, the use of PAR4 inhibitors in this patient population seems unlikely in the first instance. Rather, the focus is more likely to be on patient groups poorly served by current therapies.

(2) Patients with acute coronary syndrome are less well managed with standard-of-care therapies. Anti-platelet agents are used for both short- and long-term prevention approaches for these patients [46]. Dual therapy (aspirin and a P2Y_12_ inhibitor) is common, but associated with an increased bleeding risk [47]. The reasons for the relative insensitivity of these patients to current anti-platelet drugs remain unknown. However, recent work identified that platelets from patients with coronary artery disease have a markedly increased population of thrombin-induced procoagulant platelets when compared with healthy subjects, and that this increased response was only partially suppressed by anti-platelet therapy [48]. This apparent hyper-responsiveness to PAR4-mediated platelet thrombin responses in patients with coronary artery disease suggests these patients might be particularly well-served by strategies targeting PAR4.

(3) Diabetes-induced cardiovascular complications are a huge and growing burden and remain surprisingly resistant to current anti-platelet therapies [49,50,51,52]. PAR4 has been shown to play critical roles in diabetes-related cardiovascular complications in animal models, suggesting another potential disease setting for future PAR4 inhibitors. Specifically, in a murine model of type I diabetes, the increased susceptibility to arterial thrombosis was associated with enhanced platelet PAR4 responses [53,54,55], while increased PAR4 expression has been reported in the aorta and carotid arteries of diabetic mice [53,54]. This increase in PAR4 expression may drive the enhanced neointima formation in diabetic mice, suggesting that PAR4 inhibition may provide the combined effect of both the prevention of the vascular hypertrophy, that underlies the disease, and the prevention of the thrombosis that triggers it.

(4) Cancer-associated thrombosis is the second leading cause of death in cancer patients after their malignancy. Irrespective of cancer type, measurable activation of platelets and coagulation correlates with the extent of tumour progression and negative clinical outcome. It is well established that elevated levels of platelet-derived extracellular vesicles correlate with an increased risk of thrombotic events in patients with cancers [56]. However, few studies have investigated the relationship between cancer cells and platelets. Yet PAR4 expression is up-regulated in aggressive breast cancers [56,57] and one study has shown PAR4 is the key enhancer for promoting platelets binding to SW480 colon cancer cells [56], illustrating that PAR4 could be a potential target for cancer-induced thrombosis.

(5) Finally, stroke is the second leading cause of death in the world. There is a growing interest in the involvement of PAR4 in cerebral ischemia. PAR4-deficient mice are protected from strokes induced by a transient middle cerebral artery occlusion (notably, a non-thrombotic model of thrombosis), with reduced infarct volume and significant improved neural function after cerebral injury when compared with wildtype mice [58]. Of note, the Phase 2 trial of BMS-986141 was performed in patients who had undergone mini-strokes.

## 6. What Are the Major Issues to Consider for PAR4 Inhibitors?

What are the side effects of long term, systemic PAR4 inhibition? While these are unknown, previous work across a number of model systems provide some clues. Aside from platelets, PAR4 is present in several other cells and likely plays multiple different physiological roles. For example, PAR4 is highly expressed in lung, pancreas, thyroid, testis and the small intestine [59], and to a lesser extent in skeletal muscle, the adrenal gland, placenta and prostate [59], as well as on peripheral sensory neurons, most notably in the dorsal root ganglion [60]. PAR4-deficient mice have been widely used in efforts to determine the physiological function(s) of PAR4 in these, and other, cell types. It is worth noting that PAR4-deficient mice display no major spontaneous phenotype beyond very mild perinatal bleeding [61]. However, several inducible phenotypes have been reported in PAR4-deficient mice, and PAR4 activation has been shown to have some ‘favourable’ biological effects that may be overcome by long-term pharmacological inhibition of the receptor. For example, PAR4 activation in mice has been shown to protect mice from Coxsackievirus B3 and H1N1 influenza A virus infection [62,63] and to limit bacterial growth in Streptococcus-induced pneumonia [63]. It is proposed that inhibiting PAR4 could lead to loss of protection against certain infections. In addition, the recent detection of PAR4 on various cancer cells suggests PAR4 might be involved in cancer development and cancer progression. However, there are conflicting results of the role of PAR4 in different cancer types, and any impact of systemic PAR4 inhibition remains unclear.

One system that may be most affected is the inflammatory system. Thrombin promotes inflammation, and some of these responses appear to be mediated through PAR4. For example, PAR4 activation releases pro-inflammatory cytokines in various tissue types [64,65], and drives leukocyte recruitment and adherence [66]. PAR4 activation also leads to the formation of edema in a rodent paw model mediated by leukocyte and the kallikrein-kinin system [67] and PAR4-deficient mice are protected from the edema induced by a generic stimulus in this model [68]. These findings all suggest an important role of PAR4 in inflammation. Interestingly, increased PAR4 mRNA has been detected in various tissue types in response to inflammatory stimuli [69,70,71]. Although the reasons for this remain unknown, one hypothesis is that PAR4 mediates a positive feedback loop between coagulation and inflammation.

What will be the impact, if any, of the recently identified PAR4 sequence variants on the utility of PAR4 inhibitors? Previous studies have identified single nucleotide polymorphisms (SNPs) of PAR4 that appear to impact on receptor function. The most well studied of these is rs773902, which results in a sequence variant at amino acid position 120 of PAR4 and determines if the residue is a threonine or alanine. This is a prevalent mutation (‘minor’ allele frequency between 0.3 and 0.8, depending on the population). The SNP frequency seems to be correlated to race: The Thr120 variant is markedly more frequent in a self-identified black population than a self-identified white population in a US study of 70 participants [72], while a separate study of 202 healthy Japanese showed only 5.9% Thr120 sequence variant carriers [73]. Defining race remains an issue. This is exemplified by a PAR4 genotype study conducted in a Somali population that showed distinct frequencies within tribal populations [74], and demonstrated that future studies should be cautious in identifying race or ethnicity. Perhaps most importantly, though, the rs773902 SNP seems to impact platelet function. Platelets from individuals expressing the Thr120 PAR4 sequence variant are hyper-reactive to PAR4 agonists and hypo-reactive to the small molecule PAR4 antagonist, YD-3 [72], although not the antibody inhibitor RC3 [22]. The clinical impact of this change in receptor sensitivity to agonists and/or antagonists, if any, remains unknown, although initial studies suggest some effect. The PAR4 sequence variant affects an individual’s outcome after PAR1 inhibition, with analysis of the TRACER trial for vorapaxar indicating lower rates of major bleeding in patients with the ‘hyper-reactive’ Thr120 PAR4 variant [75]. In addition to effects on PAR1, an individual’s genotype at rs773902 may also impact their response to P2Y_12_ inhibitors, with individuals expressing the Thr120 PAR4 variant less sensitive to prasugrel following the percutaneous coronary intervention [76]. Similarly, inhibition of cyclooxygenase or P2Y_12_ was less effective in individuals expressing the Thr120 PAR4 sequence variant. Here, ex vivo thrombus formation was enhanced in these individuals, regardless of the presence (or type) of anti-platelet therapy [77]. Whether or not such changes in antagonist efficacy extend to PAR4 inhibitors directly is unknown, but any variability may hinder PAR4 inhibitor development. To this end, more investigation is warranted. Further, understanding the mechanism/s behind the SNP-induced change in platelet function may be important for the design and clinical utility of PAR4 antagonists. One simple explanation is an increase in PAR4 expression on the platelet surface. Another is a constitutively active receptor. Yet another is an alteration in downstream signal coupling or changes in receptor desensitisation [75]. None of this has been investigated in great detail, but is of clear interest as PAR4 antagonists move forward in their clinical development.

## 7. Conclusions

Since the 1991 discovery of the first PAR, PAR1, there have been substantial advances in understanding the role of these receptors in platelet biology and in developing PAR inhibitors as anti-thrombotics. A more recent focus on PAR4 has led to a greater understanding of the functions of this receptor on human platelets and how this receptor’s function varies to that of PAR1. The role of PAR4 in driving the later-stages of platelet activation, most notably the platelet procoagulant function, has revealed an attractive and novel drug target. As a result, a number of distinct PAR4 inhibitors are currently in clinical development. The early evidence indicates that PAR4 inhibition will provide a greater therapeutic window than PAR1 inhibition and even standard-of-care, such as P2Y_12_ inhibition. The field awaits the further clinical development of these first-in-class agents with bated breath.

## Figures and Tables

**Table 1:** Major PAR4 inhibitors and their mode of action.

Drug Class	Antagonist	Target Site	IC_50_	Stage of Development	Key Reference
Peptidomimetic	tc-YPGKF-NH_2_	Ligand binding site *	100 µM	Tool compound	Hollenberg et al., 2004 [32]
Pepducin	P4pal-i1	First intracellular loop	≈ 5 µM	Preclinical	Leger et al., 200 [33]
	P4pal-10	Third intracellular loop	≈ 1 µM	Preclinical	Covic et al., 2002 [34]
Small molecule	YD-3	Ligand binding site *	28 µM	Tool compound	Wu et al., 2002 [35]
	BMS-986120	Ligand binding site *	8 nM	Phase I	Wong et al., 2016 [36]
	BMS-986141	Ligand binding site *	6 nM	Phase II	Wong et al., 2017 [37]
Antibody	CAN12 (rabbit polyclonal)	N-terminal anionic cluster	10 ng/mL	Tool compound	Mumaw et al., 2014 [38]
	RC3 (human monoclonal)	Thrombin cleavage site	5 µg/mL	Preclinical	French et al., 2018 [22]

* presumed site of action.
